# A national study confirms that *Escherichia coli* from Australian commercial layer hens remain susceptible to critically important antimicrobials

**DOI:** 10.1371/journal.pone.0281848

**Published:** 2023-07-07

**Authors:** Rebecca Abraham, Hui San Allison, Terence Lee, Anthony Pavic, Raymond Chia, Kylie Hewson, Zheng Zhou Lee, David J. Hampson, David Jordan, Sam Abraham

**Affiliations:** 1 Antimicrobial Resistance and Infectious Diseases Laboratory, Harry Butler Institute, Murdoch University, Murdoch, Western Australia, Australia; 2 Birling Avian Laboratories, Bringelly, New South Wales, Australia; 3 Australian Eggs, North Sydney, New South Wales, Australia; 4 Sativus Pty Ltd, Beenleigh, Queensland, Australia; 5 New South Wales Department of Primary industries, Wollongbar, New South Wales, Australia; University of Perugia: Universita degli Studi di Perugia, ITALY

## Abstract

Controlling the use of the most critically important antimicrobials (CIAs) in food animals has been identified as one of the key measures required to curb the transmission of antimicrobial resistant bacteria from animals to humans. Expanding the evidence demonstrating the effectiveness of restricting CIA usage for preventing the emergence of resistance to key drugs amongst commensal organisms in animal production would do much to strengthen international efforts to control antimicrobial resistance (AMR). As Australia has strict controls on antimicrobial use in layer hens, and internationally comparatively low levels of poultry disease due to strict national biosecurity measures, we investigated whether these circumstances have resulted in curtailing development of critical forms of AMR. The work comprised a cross-sectional national survey of 62 commercial layer farms with each assessed for AMR in *Escherichia coli* isolates recovered from faeces. Minimum inhibitory concentration analysis using a panel of 13 antimicrobials was performed on 296 isolates, with those exhibiting phenotypic resistance to fluoroquinolones (a CIA) or multi-class drug resistance (MCR) subjected to whole genome sequencing. Overall, 53.0% of isolates were susceptible to all antimicrobials tested, and all isolates were susceptible to cefoxitin, ceftiofur, ceftriaxone, chloramphenicol and colistin. Resistance was observed for amoxicillin-clavulanate (9.1%), ampicillin (16.2%), ciprofloxacin (2.7%), florfenicol (2.4%), gentamicin (1.0%), streptomycin (4.7%), tetracycline (37.8%) and trimethoprim/sulfamethoxazole (9.5%). MCR was observed in 21 isolates (7.0%), with two isolates exhibiting resistance to four antimicrobial classes. Whole genome sequencing revealed that ciprofloxacin-resistant (fluoroquinolone) isolates were devoid of both known chromosomal mutations in the quinolone resistance determinant regions and plasmid-mediated quinolone resistance genes (*qnr*)—other than in one isolate (ST155) which carried the *qnrS* gene. Two MCR *E*. *coli* isolates with ciprofloxacin-resistance were found to be carrying known resistance genes including *aadA1*, *dfrA1*, *strA*, *strB*, *sul1*, *sul2*, *tet(A)*, *bla*_TEM-1B_, *qnrS1* and *tet(A)*. Overall, this study found that *E*. *coli* from layer hens in Australia have low rates of AMR, likely due to strict control on antimicrobial usage achieved by the sum of regulation and voluntary measures.

## Introduction

Resistance amongst bacteria to high priority, critically important antimicrobials (CIAs)—such as extended-spectrum cephalosporins, fluoroquinolones, carbapenems, and colistin—threatens the therapeutic options for treatment of severe infections in humans [1]. Emergence of CIA-resistant bacteria in food-producing animals further exacerbates this risk, either due to bacteria (commensals or pathogens) or genetic determinants being transferred via food, or via environmental pathways to infiltrate human microflora. Globally, there have been increasing reports of CIA-resistant indicator bacteria such as *Escherichia coli* isolated from food-producing animals. In 2020, the Danish Integrated Antimicrobial Resistance Monitoring and Research Programme (DANMAP) reported a 16% prevalence of fluoroquinolone-resistant *E*. *coli* in broiler chickens [2] while a recent French study reported a 16.5% prevalence of colistin-resistant *E*. *coli* in veal calves [3]. Moreover, in 2019 the American National Antimicrobial Resistance Monitoring System (NARMS) reported a 3.5% prevalence of fluoroquinolone-resistant *E*. *coli* in pigs which was the highest reported prevalence to date in the United States [4].

The Australian livestock sector is an exception to the trend for increasingly prevalent CIA colonisation of food-animals [5–10]. This favourable antimicrobial resistance (AMR) status has been attributed to Australia’s isolated geographic location, longstanding regulatory constraints on the use of CIAs (such as extended-spectrum cephalosporin, fluoroquinolone and colistin) in food-producing animals [11], and strict quarantine conditions at the national border [12]. Some sectors also achieve avoidance of antimicrobials by virtue of both the limited impact of bacterial disease and the constrained availability of antimicrobials as part of the regulatory process for eliminating chemical residues from animal products. An example of the latter is the commercial layer hen industry where for much of a hen’s existence, they are continuously producing eggs for human consumption and therefore must be minimally exposed to medications, including antimicrobials, that might accumulate residues in eggs.

Eggs from the domestic fowl are one of the most heavily consumed animal products worldwide. In Australia, there has been strong growth in consumer demand for eggs over the past decade. In 2021 it was estimated that on-average 246 eggs were consumed per person annually [13]. This increasing level of egg consumption, has been linked to increases in foodborne illness outbreaks, often associated with sub-optimal handling of eggs in food establishments [14]. Further, the use of poultry manures as fertiliser on horticultural crops can potentially produce other avenues for foodborne illness in humans [15]. To improve the clarity of the AMR risks from the egg industry to the community, surveillance of the AMR status of layer flocks is needed. The most recent survey investigated AMR presence in *Salmonella* [16], considered in the context of previous data [17], revealed overall low rates of resistance to all tested antimicrobials. However, for the purpose of comparison, and for establishing a baseline description of AMR in the layer hen sector, a comprehensive evaluation of the AMR status of *E*. *coli* in layer hens is needed. The objective of this study was to perform a national cross-sectional study to define the AMR carriage among commensal *E*. *coli* isolates from commercial layer hens. In addition, genomic characterisation of isolates expressing resistance to CIA were undertaken to support inferences on the epidemiological significance of the findings. We hypothesised that *E*. *coli* from Australian layer hens would display low rates of AMR due to longstanding restrictions on antimicrobial use within the Australian livestock sector including special constraints applied to antimicrobial use for Australian layer hens, and the low levels of AMR detected in previous surveys.

## Materials and methods

### Sample collection and approach

Cloacal swabs (n = 296) were collected from healthy commercial table egg laying chickens on 62 enrolled farms between August 2019 and January 2020. Informed consent to participate in this study was provided verbally by all enrolled farms. Five swabs were collected under veterinary supervision from each production unit, defined by management system (e.g. caged, free-range egg production systems). A commercial entity was eligible for inclusion in this survey if they had at least one commercial egg production unit. Swabs were transported on ice and processed within 24 hours of collection. This work was approved by Murdoch University Ethics committee (Permit number: Cadaver 944).

### *E*. *coli* isolation

Upon arriving at the laboratory, swabs were vortexed in 10 mL of buffered peptone water followed by direct streaking onto *E*. *coli* chromogenic agar (CHROMagar^™^ ECC, Edwards Australia). All agar plates were incubated at 37°C for 18 hours. One presumptive *E*. *coli* colony from each plate was selected based on chromogenic reaction (as detailed by the manufacturer) and sub-cultured onto CHROMagar^™^ Coli ID agar (Edwards Australia) for purity. *E*. *coli* isolation was confirmed using an indole test followed by MALDI-TOF mass spectrometry (Microflex, Bruker, MA, USA). Confirmed *E*. *coli* isolates were frozen at -80°C in 1 mL of Luria-Bertani broth with 20% glycerol.

### Antimicrobial susceptibility testing

Prior to antimicrobial susceptibility testing, isolates were recovered onto Columbia sheep blood agar (Edwards Australia) and checked for purity. The susceptibility of the isolates to antimicrobials was determined by broth microdilution according to the Clinical and Laboratory Standards Institute (CLSI) ISO 20776 standards [18]. Drug panels were prepared in-house using a customised Freedom EVO genomics platform [19]. Inocula were prepared manually at 0.5 McFarland standard as per CLSI guidelines and dispensed using the Sensititre AIM^ࡊ^ Automated Inoculation Delivery System. After incubation at 37°C for 18 hours, images of the assay plates were taken and read using the Sensititre^ࡊ^ Vizion ^ࡊ^ Digital MIC Viewing System with the minimal inhibitory concentration identified based on CLSI guidelines. *E*. *coli* ATCC 25922 was used as the control strain. Susceptibility towards thirteen antimicrobials representing ten classes were assessed and includes beta-lactam (amoxicillin/clavulanic acid and ampicillin), second-generation cephalosporin (cefoxitin), third-generation cephalosporin (ceftiofur and ceftriaxone), phenicol (chloramphenicol and florfenicol), quinolone (ciprofloxacin), polymyxin (colistin), aminoglycoside (gentamicin and streptomycin), tetracycline (tetracycline) and folate pathway inhibitor (trimethorprim/sulfamethoxazole).

Interpretation of susceptibility was based on The European Committee on Antimicrobial Susceptibility Testing (EUCAST) epidemiological cut-off (ECOFF) breakpoints which categorise isolates as wild type or non-wild type [20] and differs from CLSI clinical breakpoints that determines whether isolates are clinically resistant to the antimicrobial used for clinical treatments [21]. ECOFF breakpoints were chosen due to non-wild type isolates having been shown to contain AMR genes even with MIC values below the defined CLSI breakpoint [22]. Therefore, isolates categorised as wild type and non-wild type were classified as susceptible and resistant respectively. Multi-class drug resistance (MCR) was classified by ECOFF for isolates exhibiting non-wild type phenotype to three or more antimicrobial classes (≥ three classes of antimicrobials).

### Whole genome sequencing

Whole genomic analysis was undertaken for any *E*. *coli* isolates exhibiting resistance to CIAs. DNA extraction was performed on the isolates using the MagMAX Multi-sample DNA extraction kit (ThermoFisher Scientific) as per the manufacturer’s instructions. DNA library preparation was performed using a Celero^™^ DNA-Seq Library Preparation Kit (NuGen, Tecan) as per the manufacturer’s recommendations and then was sequenced on the Illumina Nextseq platform [23].

### Bioinformatics analysis

Sequencing files were assembled using SPAdes denovo assembler (v3.14.0) [24]. Antimicrobial resistance genes were identified using ABRicate (v0.8.7) with default settings. The sequence types (STs) of the sequenced isolates were established by *in silico* multilocus sequence typing as previously described [23].

## Results

### Phenotypic antimicrobial resistance

**Fig 1:**
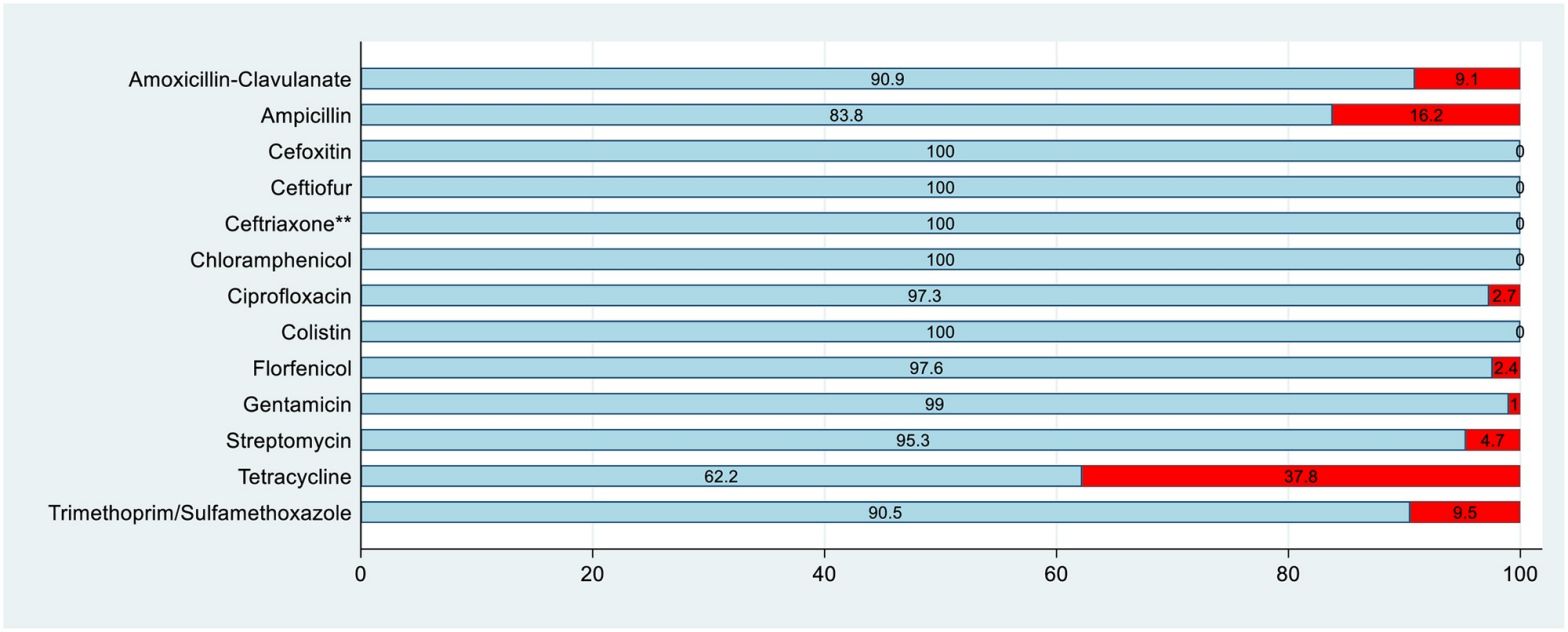
Antimicrobial resistance patterns for *E*. *coli* (*n* = 296) based on EUCAST epidemiological cut-off (ECOFF) breakpoints. The percentage of susceptible isolates is marked in blue and the percentage of resistant isolates is marked in red, unless otherwise indicated by footnotes (Percentage results were rounded to one decimal place). ** Data for this antimicrobial was based on CLSI clinical breakpoints due to the ECOFF breakpoint being below the dilution range.

In total, 296 *E*. *coli* isolates were recovered from birds on 62 farms. The majority of isolates were susceptible to all antimicrobials tested (*n* = 157, 53.0%), and all isolates were susceptible to cefoxitin, ceftiofur, ceftriaxone, chloramphenicol and colistin (Fig 1, Table 1). Resistance was observed for amoxicillin-clavulanate (9.1%), ampicillin (16.2%), ciprofloxacin (2.7%), florfenicol (2.4%), gentamicin (1.0%), streptomycin (4.7%), tetracycline (37.8%) and trimethoprim/sulfamethoxazole (9.5%) (Table 1). Of the eight isolates classified as non-wild type for ciprofloxacin (fluoroquinolone, CIA) (Table 1), none were also above the clinical breakpoint of 1 mg/L [21].

**Table 1:** Distribution of minimum inhibitory concentrations (mg/L) for *Escherichia coli* isolates (*n* = 296) from faeces of Australian layer chickens. The percentage of resistance based on ECOFF breakpoints (non-wild type) and clinical breakpoints (clinical resistance) are presented on the right-hand side (Percentage results were rounded to one decimal place). Vertical bars indicate position of ECOFF breakpoint.

	Minimum Inhibitory Concentration (μg/ml)																		
drug	0.016	0.03	0.06	0.13	0.25	0.5	1	2	4	8	16	32	64	128	256	nw*	nw_ci^	cr^#^	cr_ci^
Amoxicillin-Clavulanate							2.4	8.4	39.2	40.9	6.4		2.7			9.1	6.1,13	2.7	1.2,5.3
Ampicillin							.7	10.5	54.1	18.6	3.7	3	9.5			16.2	12.2,20.9	12.5	9,16.8
Cefoxitin							3.4	43.9	40.2	11.1	1.4					0	0,1.2	0.0	0,1.2
Ceftiofur				20.3	60.1	18.6	1									0	0,1.2	0.0	0,1.2
Ceftriaxone					95.9	3.7	.3									.	.	0.0	0,1.2
Chloramphenicol								1	24.3	67.9	6.8					0	0,1.2	0.0	0,1.2
Ciprofloxacin	97	.3		1.4	1.4											2.7	1.2,5.3	0.0	0,1.2
Colistin				5.4	80.1	13.5	1									0	0,1.2	0.0	0,1.2
Florfenicol									4.4	49.7	43.6	2.4				2.4	1,4.8	45.9	40.2,51.8
Gentamicin					36.5	44.3	13.9	4.4	.7			.3				1	.2,2.9	0.3	0,1.9
Streptomycin								33.8	50.3	9.8	1.4	1.4	2	1.4		4.7	2.6,7.8	.	.
Tetracycline									59.8	2.4	2	2	33.8			37.8	32.3,43.6	37.8	32.3,43.6
Trimethoprim/Sulfamethoxazole				87.8	1.7	1	.3			9.1						9.5	6.4,13.4	9.1	6.1,13

*nw—percent non-wildtype based on ECOFF breakpoints,

^#^cr—percent clinically resistant based on CLSI guidelines,

^ci—95% confidence interval.

Grey shading represents the concentration range tested. Values outside of the shaded area indicate isolate growth at all concentrations tested.

### Multi-class drug resistance profiles for *E*. *coli*

Twenty-one (7.0%) *E*. *coli* isolates were identified as being MCR, with seven MCR profiles identified (Table 2). The most common MCR profile was towards beta-lactams, folate pathway inhibitors and tetracyclines (*n* = 10, 3.4%). Two isolates demonstrated resistance towards four classes of antimicrobials although with different MCR profiles. One (0.3%) had a profile with resistance to aminoglycosides, beta-lactams, folate pathway inhibitors and tetracyclines while the other (0.3%) had a profile with resistance to aminoglycosides, folate pathway inhibitors, quinolones and tetracyclines.

**Table 2:** Class-based antimicrobial susceptibility profiles of *E*. *coli* isolates (*n* = 296) obtained from Australian layer chickens.

phenotype	n	%
0: nil	157	53.0
1: ami	2	0.7
1: bla	11	3.7
1: fpi	3	1.0
1: phe	4	1.4
1: qui	6	2.0
1: tet	53	17.9
2: ami_tet	6	2.0
2: bla_phe	1	0.3
2: bla_tet	22	7.4
2: fpi_tet	10	3.4
3: ami_bla_tet	3	1.0
3: ami_fpi_tet	3	1.0
3: bla_fpi_tet	10	3.4
3: bla_phe_tet	2	0.7
3: bla_qui_tet	1	0.3
4: ami_bla_fpi_tet	1	0.3
4: ami_fpi_qui_tet	1	0.3

Phenotype based on ECOFF breakpoints. ami = aminoglycosides, bla = beta-lactams, fpi = folate pathway inhibitors, phe = phenicols, qui = quinolones, tet = tetracyclines

### Genomic characterisation of critically important antimicrobial resistant *E*. *coli*

Whole genome sequencing was undertaken on all eight *E*. *coli* isolates exhibiting phenotypic resistance to fluoroquinolones (CIA), two of which showed MCR phenotypes (Table 3). Quinolone-resistance determining region (QRDR) mutations or AMR genes for fluoroquinolone resistance were only detected in one isolate that carried *qnrS1*, a known plasmid-mediated quinolone-resistance gene (PMQR) [25]. The two MCR *E*. *coli* isolates harbouring phenotypic quinolone resistance belonged to two different sequence types (ST746 and ST155) and carried AMR genes to first-line antimicrobials such as aminoglycosides and tetracyclines. As shown in Table 3, not all phenotypically detected resistance was confirmed by the detection of corresponding AMR genes or mutations.

**Table 3:** Phenotype and genotype data for quinolone-resistant *E*. *coli* isolates.

MLST	Phenotype	Genotype	Isolates (n)
ST746	ami, fpi, qui, tet	*aadA1*, *dfrA1*, *strA*, *strB*, *sul1*, *sul2*, *tet(A)*	1
ST155	qui	*lnu(C)*	1
ST3714	qui	*sul1*	1
ST155	bla, qui, tet	*blaTEM-1B*, *qnrS1*, *tet(A)*	1
ST155	qui	-	2
ST355	qui	-	2

Phenotype based on ECOFF breakpoints. ami = aminoglycosides, bla = beta-lactams, fpi = folate pathway inhibitors, qui = quinolones, tet = tetracyclines

## Discussion

This study demonstrated that there is an overall low level of AMR in *E*. *coli* isolated from Australian layer hens compared to other countries [26–31], with more than half of all isolates (53.0%) being susceptible to all tested antimicrobials, and with low levels (2.7%) or absence of resistance to CIAs (fluoroquinolone, extended-spectrum cephalosporin, polymixin). MCR phenotypes were observed only among a small number of *E*. *coli* isolates (7.0%), with two isolates exhibiting resistance to four antimicrobial classes. Where phenotypic results suggested resistance, this was not always supported by the presence of known resistance genes, as previously described by other studies [32, 33], except in a single isolate that harboured a known PMQR gene. Overall, the findings are consistent with other recent Australian studies demonstrating low levels of resistance to CIAs among *E*. *coli* isolated from Australian livestock [9, 17, 34].

As layer hens are often excluded from national AMR surveillance systems (unlike broiler chickens), there has been a relative paucity of drug susceptibility data for *E*. *coli* from this class of livestock. However, comparison of findings from the current study with recent international AMR studies in layer hens shows an overall lower level of *E*. *coli* resistance among Australian layer hens compared to other countries. For example, ampicillin-resistant *E*. *coli* was only detected among 16.2% of isolates from this current study compared to 97.8%, 83.0%, 81.0%, 54.0% and 49.5% for Bangladesh [30], China [31], Portugal [29], Zambia [28] and Tanzania [27] respectively, while tetracycline-resistant *E*. *coli* was found among 37.8% of isolates from this current study compared to 89.7%, 87.3%, 62.0%, 54.3% and 53.6% for Bangladesh [30], China [31], Portugal [29], Zambia [28] and Austria [26] respectively. This also extends to CIA-resistant *E*. *coli* where the current study demonstrated low levels or absence of CIA-resistant *E*. *coli* among Australian layer hens compared to China [31], Tanzania [27], Austria [26] and Portugal [29]. Examples include the absence of cefoxitin-resistance compared to Austria (56.0%), low levels of ciprofloxacin-resistance (2.7%) compared to Portugal (59.5%), China (45.8%), Tanzania (33.8%) and Zambia (25.4%), and absence of colistin-resistance compared to China (4.9%) and Austria (73.7%). Additionally, the low level of *E*. *coli* resistance in layer chickens from this current study was consistent with Australian studies investigating the level of *Salmonella* resistance among Australian eggs and layer chickens [10, 16, 17].

Some resistance genes encountered in this study were directed against antimicrobials that are not approved for use in Australian layer hens (eg. fluroquinolones). This phenomenon reflects the labile nature of resistance in *E*. *coli* populations arising from an acquisition of resistance by gene-transfer and co-selection, adaptations allowing survival in the environment between host colonisation events, and ability to colonise multiple host species of which some are highly mobile and can spread resistance between populations. The findings in this respect are consistent with some other recent studies demonstrating low levels of AMR to high or medium importance antimicrobials among *E*. *coli* isolated from Australian cattle and meat chickens [8, 35]. In addition, there is increasing empirical data revealing cross-species transfer of resistant *E*. *coli* from humans or other animals such as pigs [36], wild birds [37, 38] and poultry [39] regardless of which antimicrobials the recipient species are exposed to [23]. It is possible that these transmission pathways have resulted in layer hens being exposed to and colonised with resistant bacteria. Another finding that requires explanation is the lack of both chromosomal QRDR mutations and PMQR (*qnr*) genes among all but one of the ciprofloxacin-resistant isolates. The lack of resistance based on clinical breakpoints indicate either MIC drift or the insensitivity of ECOFF breakpoints to distinguish wild type versus non-wild type phenotypes. Future investigations should consider the prevalence of resistance to antimicrobials of animal health significance: this will ensure One Health approaches to AMR are implemented to avoid unintended animal health consequences from only focusing on antimicrobials of importance to human health. Continued work on minimising flock disease and adopting alternative therapies that do not require antimicrobial use also is required.

In conclusion, this study reveals low rates of overall resistance including the absence of significant resistance to antimicrobials of high importance amongst commensal *E*. *coli* isolated from Australian layer hens. This supports similar findings from recent studies of Australian cattle, pigs and meat chickens [8–10, 16, 35, 40]. The low rates are likely due to decades of stringent regulatory control on antimicrobial use, biosecurity measures, and infection prevention practises within the Australian egg industry and livestock [9, 41–45].
